# Flavonoids-Enriched Vegetal Extract Prevents the Activation of NFκB Downstream Mechanisms in a Bowel Disease In Vitro Model

**DOI:** 10.3390/ijms25147869

**Published:** 2024-07-18

**Authors:** Paolo Corbetta, Elena Lonati, Stefania Pagliari, Mario Mauri, Emanuela Cazzaniga, Laura Botto, Luca Campone, Paola Palestini, Alessandra Bulbarelli

**Affiliations:** 1 School of Medicine and Surgery, University of Milano-Bicocca, Via Cadore 48, 20900 Monza, Italy; p.corbetta@campus.unimib.it (P.C.); mario.mauri@unimib.it (M.M.); emanuela.cazzaniga@unimib.it (E.C.); laura.botto@unimib.it (L.B.); paola.palestini@unimib.it (P.P.); alessandra.bulbarelli@unimib.it (A.B.); 2 Bicocca Center of Science and Technology for Food, University of Milano-Bicocca, Piazza della Scienza 2, 20126 Milano, Italy; luca.campone@unimib.it; 3 ZooPlantLab, Department of Biotechnology and Biosciences, University of Milano-Bicocca, Piazza della Scienza 2, 20126 Milano, Italy; stefania.pagliari@unimib.it

**Keywords:** IBD, functional food, vegetal extract, inflammation, in vitro model of the intestinal barrier, NF-κB pathway, pro-inflammatory cytokines, flavones

## Abstract

Inflammatory bowel disease (IBD) incidence has increased in the last decades due to changes in dietary habits. IBDs are characterized by intestinal epithelial barrier disruption, increased inflammatory mediator production and excessive tissue injury. Since the current treatments are not sufficient to achieve and maintain remission, complementary and alternative medicine (CAM) becomes a primary practice as a co-adjuvant for the therapy. Thus, the intake of functional food enriched in vegetal extracts represents a promising nutritional strategy. This study evaluates the anti-inflammatory effects of artichoke, caihua and fenugreek vegetal extract original blend (ACFB) in an in vitro model of gut barrier mimicking the early acute phases of the disease. Caco2 cells cultured on transwell supports were treated with digested ACFB before exposure to pro-inflammatory cytokines. The pre-treatment counteracts the increase in barrier permeability induced by the inflammatory stimulus, as demonstrated by the evaluation of TEER and CLDN-2 parameters. In parallel, ACFB reduces p65NF-κB pro-inflammatory pathway activation that results in the decrement of COX-2 expression as PGE2 and IL-8 secretion. ACFB properties might be due to the synergistic effects of different flavonoids, indicating it as a valid candidate for new formulation in the prevention/mitigation of non-communicable diseases.

## 1. Introduction

Nowadays, people are exposed to several environmental stressors, such as air pollution, chemical stressors, junk food and unhealthy diets, as well as a frenetic daily life that affects physical and mental health. Therefore, in Western countries, stress-related diseases are increasing with an important economic, social and healthcare burden. Among pathologies called non-communicable diseases (NCDs), the incidence of inflammatory bowel disease (IBD) is increasing worldwide, becoming a global problem. Its prevalence is rising in every country, including the newly industrialized Asia, South America and the Middle East [1]. IBD is a group of debilitating auto-immune disorders characterized by chronic inflammation and immunological dysregulation [2,3]. The main diseases belonging to this group are Crohn’s disease (CD) and ulcerative colitis (UC), which affect the gastrointestinal tract differently but present common signs and symptoms: abdominal pain, diarrhea, fatigue, weight loss and bloody stools. The onset of the pathology is defined by an alternation of active and quiescent phases accompanied by a progression of structural damage to the bowel and crypt injuries that eventually lead to a cumulative chronic condition [4,5]. Several components interplay in the complex etiology of IBD, from genetic factors to environmental stressors, until the gut microbiota dysbiosis. Nevertheless, inflammation is central in the pathobiology [6]; thus, the therapies aim to achieve and maintain remission using diverse anti-inflammatory approaches, including immunomodulators, steroids, monoclonal antibodies direct against TNF-α, a pro-inflammatory cytokine enriched in IBD patient plasma and surgical interventions [7,8]. Nonetheless, long-term remission is still a challenge because the current treatments are not sufficient to achieve the goal (almost 30% of patients are non-responders to anti-TNFα) and are often related to adverse effects [6]. Therefore, complementary and alternative medicine (CAM) is becoming more and more diffuse practice among many IBD patients (about 20–60%) to use together with conventional treatments [9]. Nutritional strategies are the most common CAM employed by patients, since several observations indicate that a selected diet may prolong the remission state of the pathology [10]. In addition, probiotic supplementation in combination with traditional medicine is effective in IBD treatment [11]. The interaction between food and gut microbiota could be a key event in the modulation of inflammatory processes, since the impairment of host–microbiota commensalism balance is a co-cause in IBD development [12]. Recent evidence shows that a Western diet, enriched in high-energy and high-fat intake or high-fructose consumption, affects the intestinal barrier permeability, as well the gut microbiota health, while the consumption of a Mediterranean diet (MD), rich in fruit, vegetables, legumes and unrefined cereals, protects against the barrier damages in order to maintain an adequate nutritional status [13]. Fruits and vegetables are enriched in polyphenols, molecules that exert anti-inflammatory, antioxidant and immunomodulatory activities [14,15,16]. For that reason, the most recent dietary research is focused on dietary polyphenols, not only for their well-known in vitro antioxidant activity as scavengers but, above all, for their ability to interplay with signaling cascades and gene transcription [17]. Indeed, they reduce the expression of pro-inflammatory genes like multiple cytokines, lipoxygenase, nitric oxide synthase, cyclooxygenase and regulate the pro-inflammatory pathways starting from the NF-kB transcription factor, the key component of the inflammatory cascade [18]. Among others, luteolin, chrysin, quinic acid and apigenin could be involved in the regulation of intestinal permeability and inflammatory mechanisms. Each of them seems to be able to counteract the increment and/or secretion of pro-inflammatory mediators (COX-2; e.g., IL-8, NF-kB, iNOS, etc.) [17,19,20,21], suggesting an anti-inflammatory efficacy useful against IBD progression. Four flavone O-glucosides identified as glycoside derivatives of apigenin and luteolin, of quinic acid and derivatives, and also of chrysin derivatives were recently detected in a new vegetal extract obtained by a blend of three different plants: artichoke, caigua and fenugreek (ACF) [22]. Considering the ACF blend (ACFB) polyphenol content and the higher synergistic efficacy of the blend with respect to the single plant extract [22], we hypothesize an anti-inflammatory activity of the blend, which might be applied in functional food formulation. Therefore, here, we show the efficacy of ACFB in an in vitro model of IBD. Although primary human enterocyte biopsy should be the best choice for resembling the pathological conditions, it presents several disadvantages, such as difficult availability, interdonor variability and a limited timeframe in which it maintains differentiation. This study was conducted in a model consisting of Caco-2 epithelial cells differentiated and polarized on a transwell system that is the most widely used option for studies about vegetal extracts’ analysis of efficacy and safety.

## 2. Results and Discussion

### 2.1. Evaluation of Digested ACF Blend Extract’s Effects on Both Enterocyte Viability and Paracellular Permeability in the IBD Intestinal Barrier Model

Frigerio and colleagues demonstrated the bioactivity of ACFB extract on cholesterol balance. In that studio, the chemical analysis of ACF extract revealed the presence of several polyphenols with anti-inflammatory activity [22]. The UHPLC-DAD-HRMS/MS analysis of the digested ACFB extract showed the presence of most of the molecules identified by Frigerio et al. and also good resistance to the gastro-intestinal digestion process (Table 1 and Supplementary Figure S1).

**Figure 1:**
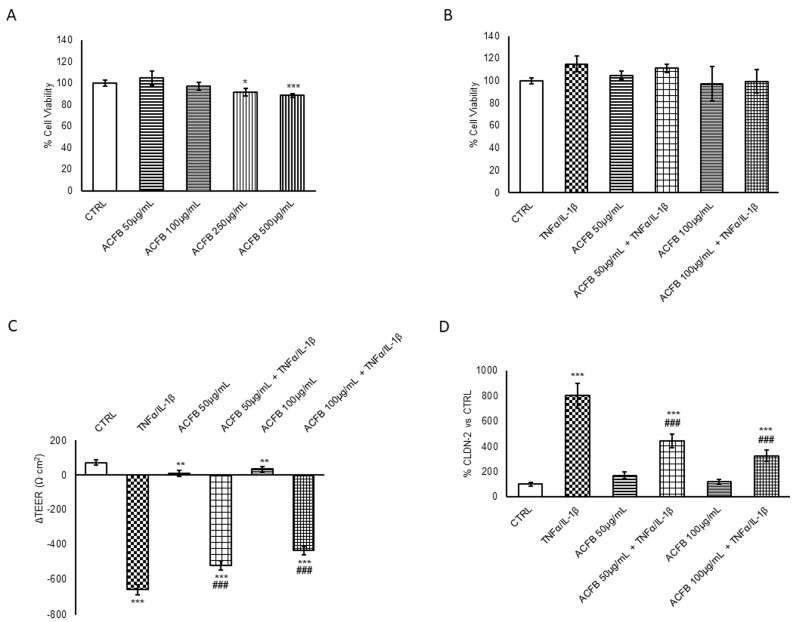
Cell viability assay and barrier integrity evaluation. (**A**) Caco-2 cells were treated with growing concentrations (25–250 μg/mL) of digested ACF blend for 48 h, and cell viability was evaluated by MTT assay. (**B**–**D**) Caco-2 cells were cultured on Transwell inserts for 21 days and then exposed to pro-inflammatory cytokines (TNFα 10 ng/mL + IL-1β 5 ng/mL) for 24 h after a 24 h pre-treatment with selected concentrations of digested ACFB extract (50–100 µg/mL). (**B**) MTT assay was performed, and results are reported in the histograms. Single conditions were also tested. Data are expressed as a percentage with respect to the untreated cell line used as a control and are shown as mean ± S.E. from three independent experiments. (**C**) TEER measures (Ω·cm^2^) were performed before (0 h) and after (24 h) cytokines’ treatment. The ΔTEER of cells exposed to cytokines was calculated and reported in the histograms as a number. Data represent the mean ± SEM from at least three independent experiments. (**D**) Cell lysates were harvested after different treatments. Equal amounts of homogenate samples (as 25 µg protein) were analyzed by SDS-PAGE electrophoresis and Western blotting. Claudin-2 was detected with a specific antibody and revealed by enhanced chemiluminescence (ECL). Samples were normalized on β-actin immunoreactivity. Histograms, obtained from at least three distinct experiments, represent the percentage of protein levels with respect to untreated cells as mean ± S.E. Statistical significance: * *p* < 0.05, ** *p* < 0.01, and *** *p* < 0.001 vs. untreated cells; ### *p* < 0.001 vs. TNFα/IL-1β.

Therefore, it has been evaluated whether ACFB might be a good ingredient for functional foods to use in a diet designed specifically for IBD patients [23]. Firstly, the safety of digested ACFB extract was evaluated on the Caco-2 cells differentiated and polarized in the intestinal barrier model. Thus, a dose-dependent viability assay was performed from 50 μg/mL to 500 μg/mL. An MTT analysis was conducted 48 h after the treatment, revealing that none of the concentrations tested resulted in cytotoxicity; however, there is a decreasing trend in viability over a 250 μg/mL concentration (Figure 1A). Accordingly, the two lower doses assessed were chosen for the successive analysis aimed to evaluate the anti-inflammatory power of digested ACFB. 

The inflammatory acute event in IBD resembles that already described by [23]. Caco-2 cells cultured on a transwell insert were exposed to a cytokine cocktail (TNFα/IL-1β), and the treatment’s sub-lethality was confirmed (Figure 1B). Nevertheless, the inflammatory effect was demonstrated by measuring the parameters of barrier integrity. As expected, cytokine exposure significantly reduces the TEER values, leading to a leaky and permeable intestinal barrier (Figure 1C). A comparison between ΔTEER of all treatments, alone or combined, showed that the ACFB had no effect on barrier integrity in control culture conditions, while it is able to prevent the TEER loss induced by pro-inflammatory cytokine exposure for 24 h (Figure 1C). Interestingly, ACFB pre-treatment led to TEER rescue in a dose-dependent manner, with a significant peak of 35% and with the higher concentration tested.

The intestinal barrier dysfunctionality in IBD is often associated with the increase of Claudin-2, a protein belonging to tight cell-cell junctions (TJs) [24]. Since Claudin-2 forms a paracellular channel for small cations and water, its increment and redistribution favor the dysbiosis [25]. Therefore, Claudin-2 protein levels were evaluated in different experimental conditions. According to what was observed for the TEER measurement, the ACFB treatment alone did not significantly alter the protein levels of Claudin-2 compared to a control condition, while the cytokine exposure induced an increase of about 7 times. The pre-treatment with the higher concentration of digested ACFB significantly reduced about 60% of Claudin-2 protein levels with respect to an inflammatory stimulus (Figure 1D). A similar behavior was observed with cinnamon digested extract pre-treatment [26]; nevertheless, the polyphenol-composition results quite different in the vegetal extracts. However, in a recent review, Bernardi and colleagues reported that several polyphenols and phenolic catabolites are able to partially reverse the TEER decrement in a Caco-2 monolayer, suggesting that barrier integrity and Claudin-2 expression represent a pivotal target to mitigate the symptoms. It is worth noting that polyphenols might regulate the barrier integrity and TJ structure by directly or indirectly acting at different levels: (1) intraluminal level, modulating microbiota composition, mediators production, redox status, and dietary component absorption; (2) intracellular level, modulating the nuclear-factor kappa B (NF-κB) signaling, the most important mediator of inflammation and in the upregulation of the nuclear factor erythroid 2-related factor 2 (Nrf-2) antioxidant system; and (3) systemic level, maintaining the correct balance of inflammatory processes [16]. Since the studio was performed on a Caco-2 monolayer, we can speculate that the observed outcomes are principally due to an intracellular regulation of anti-inflammatory/antioxidant mechanisms. Accordingly, flavones such as apigenin, luteolin and chrysin, enriched in ACFB digested extract, were identified as modulators of several inflammatory markers and NF-κB targets (i.e., COX-2, IL-8 and other pro-inflammatory cytokine) [27].

### 2.2. Effects of ACFB Digested Extract on NF-κB p65 Activation

**Figure 2:**
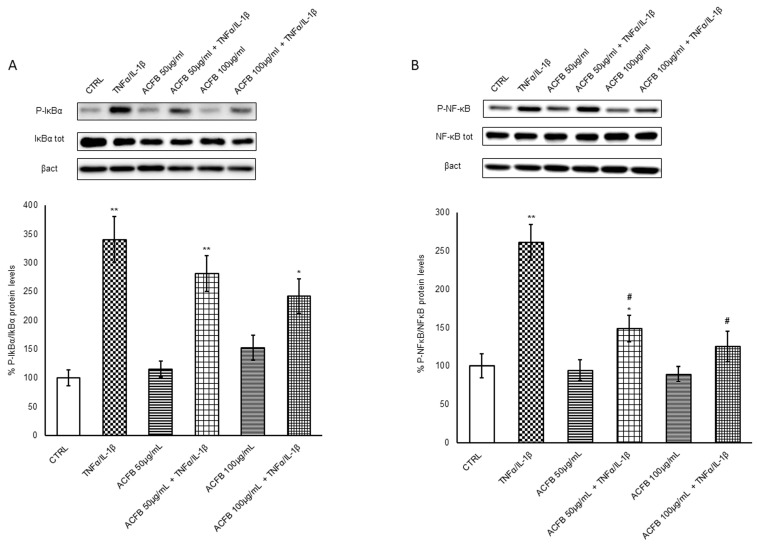
Evaluation of NF-κB activator mechanism. Caco-2 cells that were cultured on Transwell inserts for 21 days were exposed to pro-inflammatory cytokines (TNFα 10 ng/mL + IL-1β 5 ng/mL) for 24 h after a 24 h pre-treatment with selected concentrations of digested ACFB extract (50–100 µg/mL). Cell lysates were harvested, and samples were analyzed for protein concentration via bicinchoninic acid (BCA) assay. Equal amounts of homogenate samples (as protein) were analyzed by SDS-PAGE electrophoresis and Western blotting. Protein expression and phosphorylation of NF-κB and its inhibitor were detected via the use of specific antibodies—(**A**) anti-P-IkBα and anti-IkBα; (**B**) p-p65-NF-κB and NF-κB—and revealed by enhanced chemiluminescence (ECL). Samples were normalized on β-actin immunoreactivity. Histograms, obtained from at least three distinct experiments, represent the percentage of protein levels with respect to untreated cells as mean ± S.E. Statistical significance: * *p* < 0.05, ** *p* < 0.01 vs. untreated cells; # *p* < 0.05 vs. TNFα/IL-1β.

Considering the huge pro-inflammatory signaling regulated by the transcriptional factor NF-κB, its activation is finely regulated. Similar to other transcriptional factors, NF-κB is constitutively expressed but maintained inactive in the cytosol by binding with the inhibitor IκBα. NF-κB is a complex constituted by homo-heterodimers of p50, p52, p65 (Rel-A), c-Rel and Rel-B proteins. Under a pro-inflammatory cytokine stimulus, the NF-κBp65 release passes through the canonical activation pathway by IκBα phosphorylation and the following proteasome degradation. Accordingly, the exposure to TNF-α/IL-1β induced an increment of phosphorylated IκBα quote about 3 times versus untreated cells (Figure 2A), while the pre-treatment with ACFB-digested extract reduced the inhibitor phosphorylation in a dose-dependent manner, with a significant decrease by about 30% at the higher dose. In parallel, a decrease in total IκBα is observable, suggesting that, at least in part, the inhibitor underwent a degradation pathway. Once free, NF-κBp65 can be activated by phosphorylation on Ser536 and migrates into the nucleus. In line with IκBα phosphorylation, cytokine exposure induces a significant increment of P-NF-κB that is counteracted by the presence of ACFB-digested extract until a reduction by about 50% of phosphorylated form (Figure 2B).

**Figure 3:**
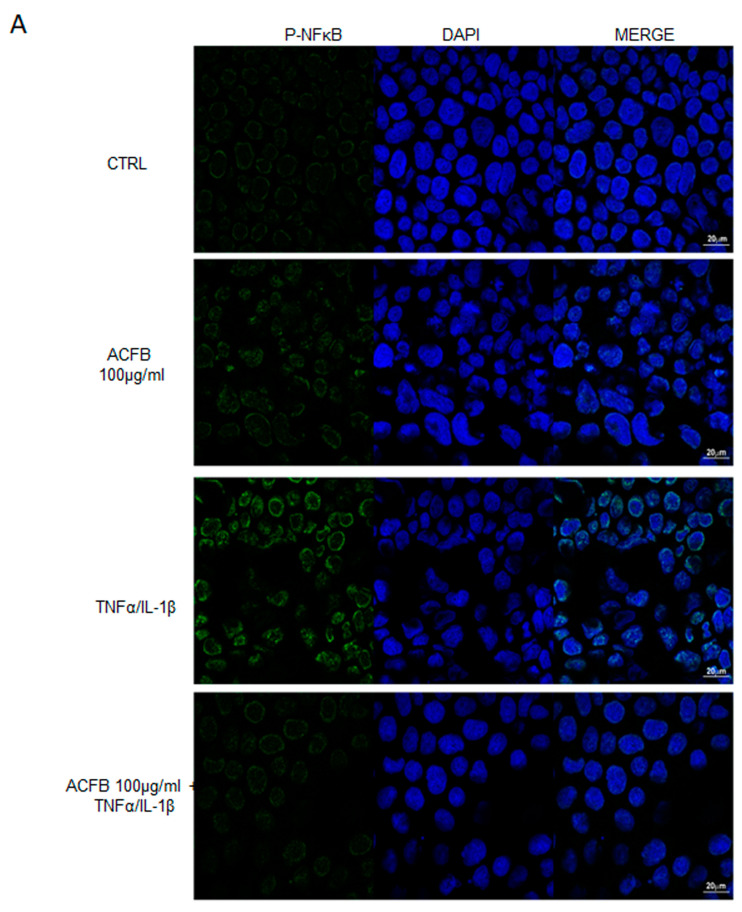

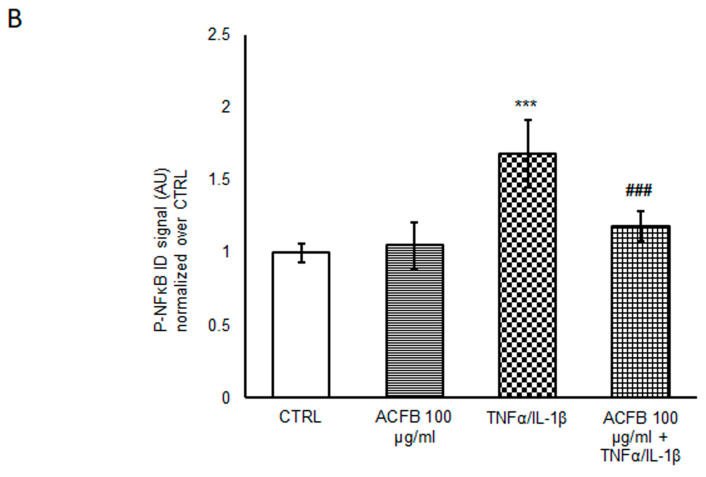
Evaluation of NF-κB localization. (**A**) Representative images of P-NFκB staining in Caco-2 cell lines treated as previously described. Green and blue channels show P-NFκB and Hoechst staining, respectively. Scale bar: 20 mm. (**B**) Histogram showing P-NFκB signal ratio detected for control vs. treated cells. Error bars represent the standard deviation of the mean derived from the acquired fields. *** *p* < 0.001 vs. untreated cells; ### *p* < 0.001 vs. TNFα/IL-1β.

As demonstrated by immunofluorescence analysis, P-NF-κB is mainly localized in the nucleus. In untreated cells (CTRL) the signal is quite low, while after the TNFα/IL-1β stimulus, the green signal is significantly increased by about 70%, and it is mainly in the perinuclear zone. Pre-treatment with ACFB 100 μg/mL alone did not significantly affect the P-NF-κB signal increase with respect to control, and it seems more diffuse in the nucleus. Instead, its presence reduces the intensity of the signal corresponding to P-NF-κB translocated into the nucleus after the pro-inflammatory stimulus (Figure 3). Hence, these results indicate that ACFB acts on intracellular pathways, preventing the enhancement of the NF-κB activator canonical pathway. The registered effect might be the result of the polyphenols mixture combination. Indeed, among the polyphenols contained in ACFB, there are mono-caffeoylquinic acids and di-caffeoylquinic acids that exert their anti-inflammatory activity by downregulating the NF-kB pro-inflammatory pathway [28]. In particular, the di-caffeoylquinic acids anti-inflammatory property was recently identified in preventing the translocation of the transcription factor NF-kB into the nucleus [29]. Consequently, NF-kB targets should be downregulated; this hypothesis was explored via the analysis of the two main inflammatory markers in IBD: the interleukin-8 (IL-8) and cyclooxygenase-2 (COX-2) enzyme.

### 2.3. ACFB-Digested Extract Prevents IL-8 Secretion Highly Induced by TNF-α/IL-1β Exposure

**Figure 4:**
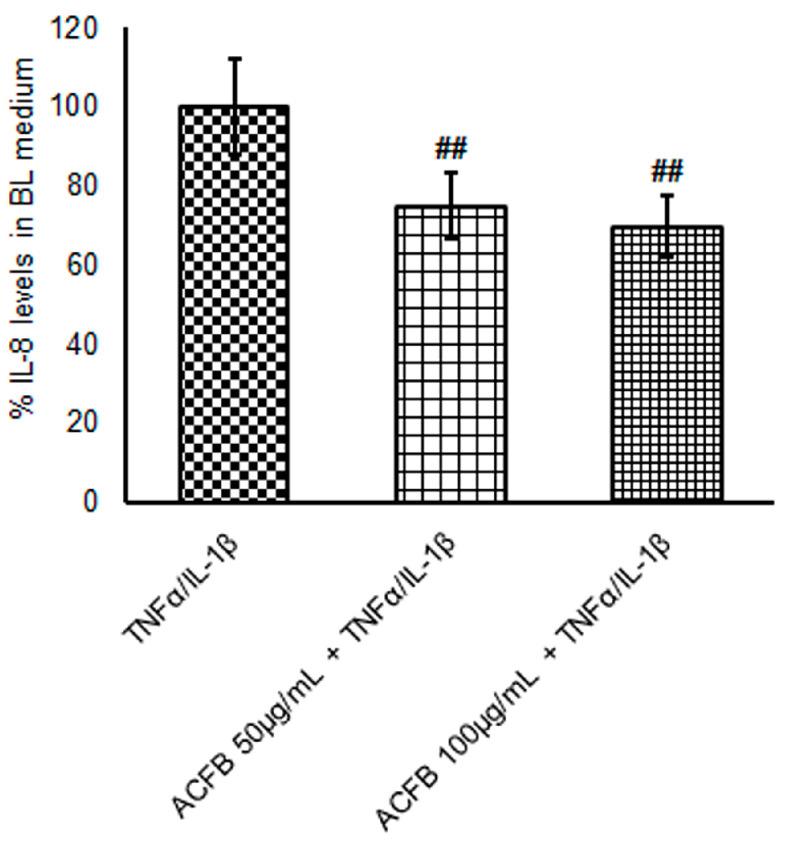
Evaluation of IL-8 secretion at the basolateral (BL) compartment. After different single or combined treatments, basolateral media were harvested. IL-8 secretion was evaluated via ELISA assay. Data were normalized by cell total protein concentration. Results reported in histograms are expressed in relative terms to inflammatory treatment and are shown as a mean ± S.E. from at least three independent experiments. Statistical significance: ## *p* < 0.01 vs. TNFα/IL-1β.

Several clinical studies demonstrated high concentrations of IL-8 in the mucosa and in the plasma of IBD patients [15], while it is not detectable in the mucosa of healthy subjects. Inflammatory mechanisms, indeed, induce the increase in this chemokine, which recruits the macrophages at the site of inflammation [28]. Here, the immune system, cells release other pro-inflammatory cytokines, worsening barrier dysfunction and the severity of inflammation. Therefore, the regulation of pro-inflammatory cytokine production and secretion could be a key mechanism for downregulating acute inflammatory burst characteristic of IBD. In this contest, ACFB seems very promising, since pre-treatment with both concentrations reduced by about 25% the huge increase of IL-8 secretion under inflammatory conditions (Table 2 and Figure 4).

Considering that, in the IBD remission phases, IL-8 concentrations are quite high in the plasma of the patients [30], a daily intake of ACFB might counteract the chronic recruitment of activated macrophages. Synergistically with di-caffeoylquinic acids, the luteolin present in the ACFB-digested extract might contribute to the protective effect on cytokine secretion. Indeed, Nunes and colleagues demonstrated that this flavone is able to reduce IL-8 levels in an in vitro model of intestinal epithelium (HT-29) exposed to a cocktail of pro-inflammatory cytokines (TNFα, IL-1β and IFN-γ) [17].

### 2.4. ACFB-Digested Extract Prevents the Cox-2/PGE2 Pathway Induced by TNF-α/IL-1β Exposure

**Figure 5:**
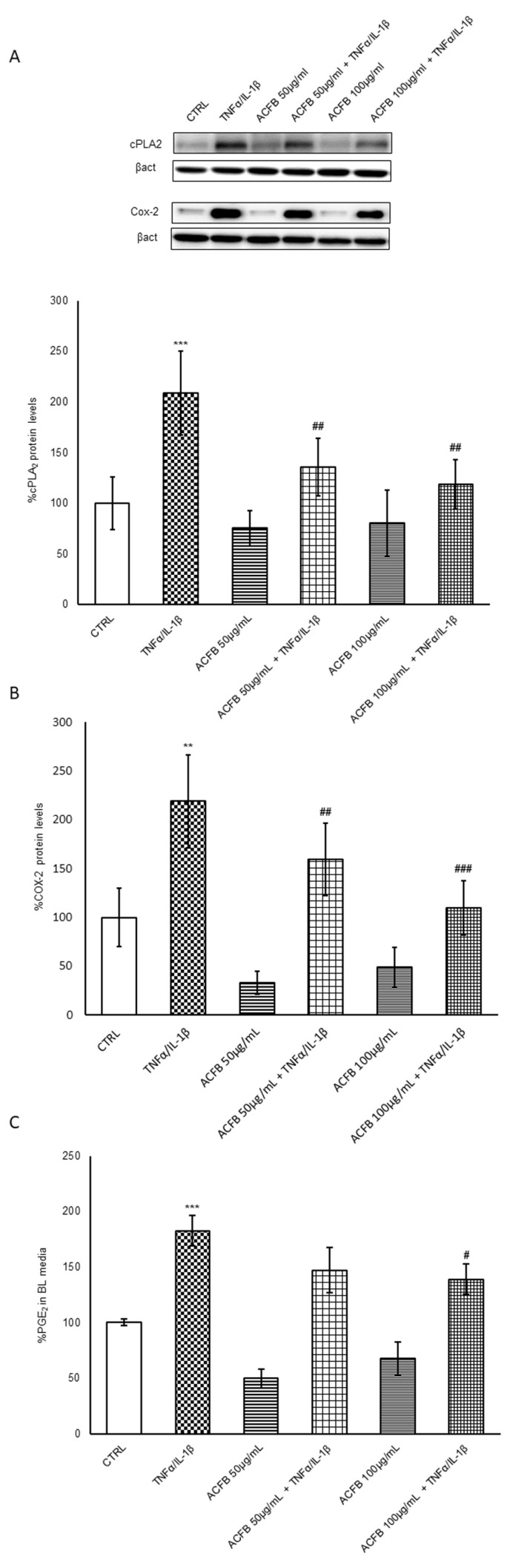
Evaluation of Arachidonic Acid metabolism. After different single or combined treatments, cell lysates and basolateral media were harvested. cPLA2 (**A**) and COX-2 (**B**) protein levels were detected with specific antibodies and revealed by enhanced chemiluminescence (ECL). Samples were normalized on β-actin immunoreactivity. Histograms, obtained from at least three distinct experiments, represent the percentage of protein levels with respect to untreated cells as mean ± S.E. (**C**) After 10 min of AA administration, PGE-2 secretion at the basolateral compartment was evaluated by an ELISA kit. Data were normalized by cell total protein concentration, and histograms are representative of at least three distinct experiments. The percentage was calculated with respect to untreated cells as mean ± S.E. Statistical significance: ** *p* < 0.01, *** *p* < 0.001 vs. untreated cells; # *p* < 0.05, ## *p* < 0.01, ### *p* < 0.001 vs. TNFα/IL-1β.

Another important inflammatory pathway active in IBD is the cyclooxygenase-2/prostaglandin E2 (PGE2). Indeed, during the acute phases of the disease, there are higher Arachidonic Acid (AA)-derived eicosanoids levels and an increased expression of enzymes related to this metabolism, such as COX-2 and 5-LOX [31]. The amount of free AA increases when released from the plasma membrane by the action of phospholipase A2 (PLA2). In particular, the cytosolic PLA2 (cPLA2) seems to play an essential role in the initiation of AA metabolism, favoring a major availability of substrate for COX-2 and other enzymes [32]. It is well known that, under pro-inflammatory stimuli, PLA2 family proteins increase [33]. Under cytokine cocktail (IL-1β/TNF-α) exposure cPLA2 doubled in Caco-2-polarized cells (Figure 5A). In parallel, COX-2 protein levels incremented by about 150% with respect to untreated cells (Figure 5B). 

Interestingly, here we show that ACFB pre-treatment is able to prevent the increment of both cPLA2 and COX-2 expression in Caco-2 cells, in a dose-dependent manner, with a reduction by about 50% at the higher dose tested (Figure 5A,B). Considering that a high AA release from phospholipids could result in lipid remodeling of the plasmatic membrane, the cPLA2 decrement is an important regulatory mechanism that could be activated by ACFB.

Based on these results, ACFB might reduce the AA metabolism and the production of eicosanoids. In particular, the PGE2s are the ones mainly associated with inflammation promotion, tissue-damage enhancement and intestinal-lumen irritation [34]. These lipid mediators are slightly higher in acute phases of IBD than in remissive ones, as well as the enzymes of the AA pathway. In order to evaluate COX-2 activity indirectly by the concentration of PGE2 produced in the different experimental conditions, AA was administered to the apical side of the barrier for 10 min, and then the PGE2 amount was immediately measured in the medium. A reduction of 25% of PGE2 production and secretion was observed in cells pre-treated with ACFB with respect to those only exposed to TNF-α/IL-1β (Figure 5C). In this pathway, luteolin could be one of the key components of ACFB able to reduce COX-2 in inflammation [17]. Synergistically, apigenin might contribute to COX-2 reduction, since its efficacy was observed in different cellular types [35]. Furthermore, the presence of chrysin might also contribute to preventing COX-2 activity since it was identified as a flavone that is able to reduce PGE2 production efficiently. Luteolin and chrysin might act as superoxide and hydroxyl radical scavengers [36]. Considering that the PGE2 concentration is rather high also in the remission phase [37], a cPLA2/AA/COX-2 pathway inhibition by nutritional approaches might be an important cofactor in the treatment of chronic inflammatory disease to extend the period of remission of IBD and decreases the risk of development of colorectal cancer in these patients.

## 3. Materials and Methods

### 3.1. Vegetal-Extract Selection for ACFB Formulation

Based on precedent evaluations conducted by Frigerio and colleagues [22], the present experiments were performed using a formulation commercially known as Omeolipid produced by EPO Srl. It is a poly-extract of *C. scolymus*, *C. pedata*, and *T. foenum*-graecum, originating from the three simultaneous vegetal extractions provided by the company. See Frigerio (2021) [22] for details about sample identification by DNA barcoding, sample collection and DNA extraction.

### 3.2. UHPLC-DAD-HRMS/MS Profiles of ACFB

The identification of compounds in the extracts of ACFB was performed following the method reported by Frigerio (2021) [22]. In detail, a Waters ACQUITY UPLC system coupled to a Waters Xevo G2-XS QT of the mass spectrometer (Waters Corp., Milford, MA, USA) was used. The separation of the analytes was performed using H_2_O + 0.1% HCOOH (A) and CH_3_CN + 0.1% HCOOH (B) as the mobile phase and setting the following elution gradient: 0–2.0 min, 5–10% B; 2.0–17.0 min, 10–35% B; and 17.0–18.0 min, 35–95%, with 5 min washout (98% B) at the end of each run and 5 min equilibration before the next injection. Kinetex Biphenyl (100 mm × 2.1 mm, 2.6 μm) at 30 °C was used as the chromatographic column, and the flow rate was set at 400 μL min^−1^. The UV spectra were acquired in the 210–400 nm range, and wavelengths of 280 and 330 nm were selected to detect target analytes. The mass spectrometer was calibrated with 0.5 M sodium formate. It was used in negative and positive ionization mode to acquire a full scan, and spectra were recorded in the *m*/*z* 100–1000 range. The source parameters were an electrospray capillary voltage of 2.5 kV, source temperature of 150 °C and desolvation temperature of 500 °C. The cone and desolvation gas flows were 10 and 1000 L/h, respectively. The scan time was 0.5 s. The cone voltage was set to 60 V, and the ramp collision energies ranged from 6 to 30 V. The phenolic compounds were characterized based on the corresponding spectral characteristics (UV and MS [M-H]- spectra), accurate mass and characteristic fragmentation, and several databases (PubChem, ChemSpider, and KEGG) were consulted. MassLynx software (version 4.2) was used for instrument control and data acquisition and processing

### 3.3. INFOGEST Methods for Digestion In Vitro Simulation

The gastrointestinal digestion simulation was carried out following the INFOGEST protocol described and used in Minekeus et al. (2014) [38]. The method was already used for cinnamon extract digestion and described in previous studies [23,39]. Briefly, the ACFB extract was dissolved in water at a concentration of 20 mg/mL, and then the gastrointestinal digestion through the oral, gastric and intestinal phases was simulated. Each step was performed by preparing a specific mixture of salts and enzymes and adjusting the pH and temperature under the conditions of the respective phase. The duration of the process was 2 h for the gastric phase, 2 h for the intestinal phase and 5 min for the oral phase. According to Pineda-Vadillo (2016) [40], the product obtained at the end of the digestion process was acidified in order to precipitate the enzymes and preserve the polyphenols. Finally, protease inhibitors were added to conserve the samples.

### 3.4. Cell Cultures

The Caco-2 cell line is a continuous line of heterogeneous human epithelial colorectal adenocarcinoma cells that, upon reaching confluence, express the characteristics of enterocytic differentiation: tight junctions, microvilli and a number of enzymes and transporters that are characteristic of enterocytes [41]. Cells were grown in Minimum Essential Medium (MEM) with nonessential amino acids supplemented with 10% heat-inactivated fetal bovine serum (FBS), 2 mM L-glutamine, 100 units/mL penicillin, 100 mg/mL streptomycin and 1% sodium pyruvate and maintained in a collagen-coated 75 cm^2^ flask at 37 °C in a 5% CO_2_ atmosphere. All reagents for cell culture were from Euroclone, Pero, Italy. In order to perform the anti-inflammatory capacity experiments Caco-2 was seeded at 100,000 cell/cm^2^ onto collagen-coated Transwell^®^ polyester membrane inserts (Corning, Glendale, AZ, USA) and maintained at 37 °C in a 5% CO_2_ atmosphere for 21 days in order to reach the proper differentiation in a polarized epithelial cell monolayer, which resembles the physiological intestinal barrier. It represents the most suitable condition to study gut maintenance and defense against inflammatory mediators and the modulation of intestinal epithelial permeability. The basolateral and the apical sides of the insert represent the circulatory and luminal poles of the intestinal epithelium, respectively. Medium was replaced both in apical and basolateral chambers every 3 days, and Transendothelial Electrical Resistance (TEER) was measured every 7 days by means of EVOM EndOhm-12 chamber (World Precision Instruments, Sarasota, FL, USA) to check the formation of a reliable intestinal barrier. 

### 3.5. ACFB Treatment and Cell Viability Evaluation

Caco-2 cells plated on Transwell^®^ inserts were treated with growing concentrations (25–250 μg/mL) of digested ACF blend for 48 h. Then, an MTT assay was performed in order to select the better concentrations for the successive experiments. Briefly, the MTT [3-(4,5-Dimethythiazol-2-yl)-2,5-diphenyltetrazolium bromide] assay is based on the reduction of tetrazolium salts to colored formazan compounds that occur in metabolically active cells. Optical density was measured with a FLUOstar Omega (BMG Labtech, Ortenberg, Germany) multi-detection microplate reader at a wavelength of 570 nm.

### 3.6. Inflammatory Treatment and Evaluation of Intestinal Barrier Integrity

In order to mimic acute inflammation, Caco-2 cells were treated with a cocktail of pro-inflammatory cytokines (TNF-α 10 ng/mL + IL-1β 5 ng/mL) for 24 h via basolateral administration, as described by Lonati et al. (2023). ACFB extract was dispensed to the apical side at the selected concentrations of 50–100 µg/mL during the 24 h preceding the inflammatory stimulus. Cell viability (MTT) and TEER were evaluated as parameters of barrier functionality after inflammation in cells pre-treated or not with ACFB. TEER was measured before and after the 24 h inflammatory treatment for every insert, and the ΔTEER [Δ(Ω·cm^2^)] between the two measures was calculated and analyzed within the groups to evaluate barrier functionality following diverse treatments.

### 3.7. Electrophoresis and Immunoblotting

An equal amount (as protein) of homogenate was analyzed by SDS-PAGE electrophoresis on 10% polyacrylamide tris-glycine gels. Proteins were transferred to a nitrocellulose membrane (Amersham, GE Healthcare Europe GmbH, Milano, Italy) and revealed by immunoblotting with specific antibodies. Immunoblottings were performed using rabbit polyclonal anti-claudin-2 (1:200; 51-6100; Thermofisher Scientific™, Milano, Italy), rabbit polyclonal anti-β-actin (1:1500; A2066 Merck s.r.l.; Milano, Italy), rabbit polyclonal anti-P-p65 NF-κB (1:1000; #4764S, Cell Signaling Technology, Inc, Danvers, MA, USA) and anti-NF-κB (1:1000; #4764S, Cell Signaling), rabbit polyclonal anti-P-IkBα (1:1000; #2859T, Cell Signaling) and mouse polyclonal anti-IkBα (1:1000; #4814T, Cell Signaling), rabbit polyclonal COX-2 (1:1000; #12282, Cell Signaling) and mouse polyclonal anti-cPLA2 (1:250) (J1507, Santa Cruz Biotechnology, Inc, Dallas, TX, USA) antibodies. Immunoreactive proteins were revealed by enhanced chemiluminescence (ECL) and semi-quantitatively estimated by LAS4000 Image Station. Normalization in the same sample was carried out with respect to β-actin homogenate samples [42,43]. 

### 3.8. IL-8 ELISA Assay

In order to assess IL-8 secretion in the compartment mimicking blood circulation, extracellular media were collected from the basolateral chamber. IL-8 concentration was evaluated by means of a commercially available ELISA assay (PeproTech EC, Ltd., London, UK), according to the manufacturer’s instructions. Samples’ concentrations were quantified in pg/mL using the standard provided by the kit, after reading absorbance with a FLUOstar Omega (BMG Labtech, Ortenberg, Germany) multi-detection microplate reader at a wavelength of 405 nm and a reference wavelength of 690 nm. Then, to facilitate a comparison between groups and to understand the anti-inflammatory potential of ACF, data normalized by cell protein concentration were expressed in relative terms to the inflammatory treatment.

### 3.9. Immunofluorescence Assay

An immunofluorescence assay was performed for the purpose of observing nuclear NF-κB localization. The cells were seeded on micro cover glasses (Prestige^®^) for 7 days. After treatments, cells were washed twice with PBS and then incubated for 10 min at room temperature with MetOH prechilled at −20 °C. Then, they were washed and incubated with PBS overnight at 4 °C. Cells were incubated for 2 h at room temperature with anti-pNFKB (#3033T, Cell Signaling Technology, Inc., Danvers, MA, USA) primary antibody (1:400 dilution in GDB buffer [0.02 M sodium phosphate buffer, 0.02% triton x-100, pH 7.4, containing 0.45 M NaCl, 0.2% (*w*/*v*) bovine gelatin]), followed by staining with Alexa 488-conjugated secondary antibody (1:100 in GDB buffer) (A11008, Thermo fisher Scientific™, Milano, Italy) for 1 hour. After two washes with PBS and staining with Hoechst 33342 (Thermofisher Scientific™, Milano, Italy), coverslips were mounted on glass slides with a 90% (*v*/*v*) glycerol/PBS solution. Images were acquired using a Zeiss LSM 710 confocal laser-scanning microscope (Zeiss, Milano, Italy), using a 63×, 1.4 N/A oil-immersion objective. Laser intensities and acquisition parameters were held constant throughout each experiment. Confocal microscopy fields were analyzed using a specific homemade/designed macro with ImageJ (https://imagej.nih.gov/ij/) software (1.54b). In detail, pNFKB signal intensity was analyzed by measuring the ID and normalized over control cells cultured in DMEM. All the data obtained were derived from at least 10 fields per experimental condition (at least 300 cells per condition).

### 3.10. PGE2 ELISA Assay

In order to mimic the modulation induced by ACFB on pro-inflammatory lipid mediators released in the bloodstream, the concentration of PGE2 in basolateral media was evaluated. First Caco-2 cell culture was treated with 10 mM Arachidonic Acid for 10 min in order to enhance the PGE2 production. PGE2 concentration was evaluated by means of a PGE2 ELISA kit (Invitrogen, Vienna, Austria). The concentration was quantified in pg/mL based on the standard provided by the kit, after reading absorbance with FLUOstar Omega (BMG Labtech, Ortenberg, Germany) multi-detection microplate reader at a wavelength of 405 nm and a correction wavelength of 570 nm.

### 3.11. Statistical Analysis

All data are expressed as mean SEM (standard error of the means). The values were compared to the negative control (untreated cells) or positive control (inflammatory stimulus) using the Sidak test, following one-way ANOVA calculation. A *p*-value < 0.05 was considered to be statistically significant.

## 4. Conclusions

To our knowledge, this is the first study to investigate the anti-inflammatory properties of extracts obtained from traditional herbs such as artichoke, caigua and fenugreek combined in a unique blend. Here, we show the synergistic effect of this novel formulation, suggesting that the phyto-complex as a whole is responsible for the benefits observed with respect to the single components. Although the simulated digestion approaches with the static model (INFOGEST) present some limitations since the model is not able to reproduce the dynamic processes of human gastric digestion, this protocol is widely used due to its simplicity and reliability. The composition in the flavones and phenolic acids of ACFB is maintained before and after digestion. Our results indicate that these bioactive molecules are able to interact with and manage the cellular stress pathways, preventing inflammatory and oxidative signaling. Moreover, ACFB seems to play a role in maintaining the intestinal epithelium barrier homeostasis and functions, adding to the several dietary nutrients that exert the same effects [44]. Although these data were obtained from in vitro cellular models, they could be of interest to the new formulations by the food companies. Hence, ACFB might represent a valid candidate for a new formulation to prevent and/or mitigate non-communicable diseases, such as cancers, degenerative diseases and a wide panel of inflammatory pathologies. It would be essential to assay the whole-formulated food product by subjecting it to digestion, chemical analysis and bioactivity study. Considering the aim of this research, clinical trials with specific nutrition strategies should be mandatory for different segments of populations. 

## Figures and Tables

**Table 1:** Metabolite identification in the ABDF digested extract by UHPLC-HRMS/MS.

No.	Rt (min)	[M-H]-	Formula	Δppm	MS/MS	Compounds *
1	2.26	353.0881	C_16_H_17_O_9_	2.3	191.0551	Mono-caffeoylquinic acid isomer
2	3.27	353.0881	C_16_H_17_O_9_	2.3	191.0551	Mono-caffeoylquinic acid isomer
3	3.39	353.0881	C_16_H_17_O_9_	2.3	191.0551	Mono-caffeoylquinic acid isomer
4	4.68	593.1505	C_27_H_29_O_15_	−0.2	503.1175/473.1074/383.0759/353.0652	Apigenin-di-C-hexoside isomer
5	4.86	593.1505	C_27_H_29_O_15_	−0.2	503.1175/473.1074/383.0759/353.0652	Apigenin-di-C-hexoside isomer
6	5.34	563.1412	C_26_H_27_O_14_	2.0	503.1175/473.1074/443.0973/383.0759/353.0652	Apigenin C-hexoside-C-pentoside isomer
7	5.57	515.1169	C_25_H_23_O_12_	−4.1	353.0880/191.0550/179.0332	Di-caffeoylquinic acid isomer
8	5.91	563.1412	C_26_H_27_O_14_	2.0	503.1175/473.1074/443.0973/383.0759/353.0652	Apigenin C-hexoside-C-pentoside isomer
9	6.00	447.0933	C_21_H_19_O_11_	1.3	357.0606/327.0497	orientin
10	6.79	431.0986	C_21_H_19_O_10_	1.9	341.0652/311.0551/283.0601	Apigenin-6-C-glucoside
11	7.70	447.0930	C_21_H_19_O_11_	0.7	285.0389	cynaroside
12	7.81	461.0720	C_21_H_17_O_12_	0.0	285.0389	Luteolin-7-O-glucuronide
13	8.61	515.1169	C_25_H_23_O_12_	−4.1	353.0880/191.0550/179.0332	Di-caffeoylquinic acid isomer
14	8.98	431.0986	C_21_H_19_O_10_	1.9	269.0424	Apigenin-7-O-glucoside
15	9.17	445.0778	C_21_H_17_O_11_	1.6	269.0424	Apigenin-7-O-glucoronide
16	10.56	399.1081	C_21_H_19_O_8_	0.3	325.0703/295.0604/267.0655	Chrysin-6-C-fucopyranoside
17	11.02	415.1035	C_21_H_19_O_9_	1.4	325.0703/295.0604/267.0655	Chrysin derivate
18	12.17	399.1981	C_21_H_19_O_8_	0.3	325.0703/295.0604/267.0655	Chrysin-6-C-fucopyranoside

* Compounds were identified as reported by Frigerio et al., 2021 [22].

**Table 2:** Concentration (pg/mL) of IL-8 secreted in the basolateral media at different conditions. *** *p* < 0.001 vs. untreated cells.

	CTRL	ACFB 50 µg/mL	ACFB 100 µg/mL	TNFα/IL-1β	ACFB 50 µg/mL + TNFα/IL-1β	ACFB 100 µg/mL + TNFα/IL-1β
pg/mL	7.31 ± 3.21	4.80 ± 3.21	11.77 ± 3.81	303.69 ± 21.11	271.70 ± 11.72	256.36 ± 10.36
vs CTRL				***	***	***

## Data Availability

All related data and methods are presented in this paper. Additional inquiries should be addressed to the corresponding author.

## Supplementary Materials

The following supporting information can be downloaded at https://www.mdpi.com/article/10.3390/ijms25147869/s1.
