# Supplementary figures and images for: Flavonoids-Enriched Vegetal Extract Prevents the Activation of NFκB Downstream Mechanisms in a Bowel Disease In Vitro Model
Source: Int J Mol Sci. 2024 Jul 18;25(14):7869. doi: 10.3390/ijms25147869 (PMC11277009; doi:10.3390/ijms25147869)

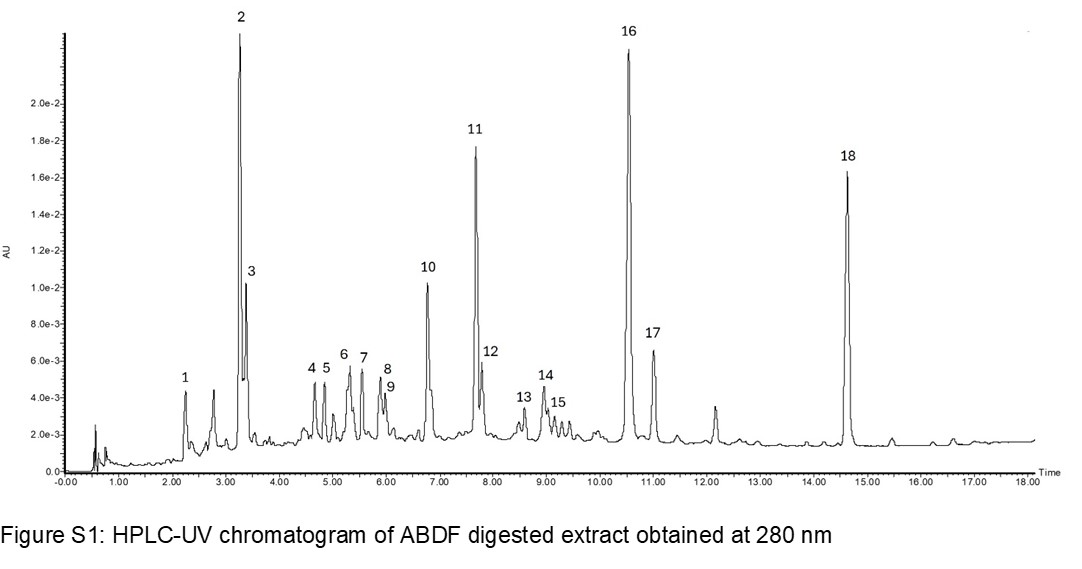

Supplement: Supplementary file 1 [file ijms-25-07869-s001.zip › ijms-3072885-supplementary.jpg]
